# Extracts from Cell Suspension Cultures of Strawberry (*Fragaria x ananassa* Duch): Cytotoxic Effects on Human Cancer Cells

**DOI:** 10.3390/molecules24091738

**Published:** 2019-05-04

**Authors:** Simona Lucioli, Fabio Pastorino, Paolo Nota, Giulia Ballan, Andrea Frattarelli, Alessia Fabbri, Cinzia Forni, Emilia Caboni

**Affiliations:** 1 Consiglio per la ricerca in agricoltura e l’analisi dell’economia agraria (CREA), Centro di ricerca Olivicoltura, Frutticoltura e Agrumicoltura (CREA-OFA), Via di Fioranello 52, 00134 Rome, Italy; paolo.nota@crea.gov.it (P.N.); andrea.frattarelli@crea.gov.it (A.F.); emilia.caboni@crea.gov.it (E.C.); 2 Laboratorio di Terapie Sperimentali in Oncologia, IRCCS Istituto Giannina Gaslini, Via G. Gaslini 5, 16147 Genoa, Italy; fabiopastorino@gaslini.org; 3 Centro Nazionale per la Salute Globale, Istituto Superiore di Sanità, Viale Regina Elena 299, 00161 Rome, Italy; giulia.ballan@libero.it (G.B.); alessia.fabbri@iss.it (A.F.); 4 Dipartimento di Biologia, Università di Roma “Tor Vergata”, Via della Ricerca Scientifica, 00133 Rome, Italy; forni@uniroma2.it

**Keywords:** plant in vitro culture, strawberry, furofurane, anticancer drugs

## Abstract

Natural compounds are emerging as agents for the treatment of malignant diseases. We previously showed that extracts from in vitro cell suspension cultures of strawberry reduced murine melanoma cell proliferation, as shown for fruit extracts. In this work, chromatographic, mass spectrometric, and spectrophotometric analyses were carried out to identify the bioactive compound exerting the detected cytotoxic activity. Moreover, aiming to confirm the anti-proliferative activity of the extracts against both paediatric and adult human tumors, cytotoxic experiments were performed on neuroblastoma, colon, and cervix carcinoma cell lines. Extracts from in vitro cell suspension cultures of strawberry induced a statistically significant reduction of cell growth in all the tumor cell lines tested. Interestingly, human fibroblasts from healthy donors were not subjected to this cytotoxic effect, highlighting the importance of further preclinical investigations. The accurate mass measurement, fragmentation patterns, and characteristic mass spectra and mass losses, together with the differences in chromatographic retention times and absorbance spectra, led us to hypothesize that the compound acting as an anti-proliferative agent could be a novel acetal dihydrofurofuran derivative (C_8_H_10_O_3_, molecular mass 154.0630 amu)

## 1. Introduction

Several natural compounds have been isolated from plant sources, and their use in the clinic leads to estimate that approximately two-thirds of the active ingredients present in anticancer drugs and in drugs for infectious diseases are of plant origin [1]. Many examples of plant-derived compounds are in phase I–II or II–III clinical trials; curcumin, ingenol mebutate, and betulinic acid, as examples, have been tested for anticancer activity [2]. However, despite the growing interest in these compounds, only a fraction of all naturally occurring chemicals have been investigated [3].

On the other hand, many natural products used for cancer treatment occur at very low levels in plant tissues and, due to their complex structure, a completely synthetic production would be too expensive. As a consequence, the challenge to produce natural compounds in a sustainable way implies that improved methods of supply of anticancer agents would be desirable. The use of plant cell cultures for the production of metabolites is an interesting area of science [4], especially when the natural supply is limited by low yields, slow growth rates, or both [5,6]. The production of metabolites via plant cell culture is renewable and environmentally friendly; the growth of in vitro plants under controlled conditions allows obtaining useful and continue quantities of metabolites.

It is already known that strawberries contain bioactive compounds able to inhibit cancer cell proliferation, as shown in several studies on human cancer cell lines. We previously showed that extracts from fruit and from cell suspension cultures of *Fragaria x ananassa* Duch (cv. Don) reduced murine melanoma cell proliferation [7,8]. Strawberry healthy effects have been attributed to the synergic activities of various nutrients and phytochemicals; however, the effect of strawberry bioactive compounds on cancer is not yet fully elucidated [9,10,11].

Paediatric neuroblastoma (NB) represents the most frequent and aggressive form of extracranial solid tumor of infants, responsible for 15% of childhood cancer deaths [12]. Among the adult population, colorectal and cervical cancers are a leading cause of cancer death worldwide [13,14]. The aim of this study was to verify the cytotoxic activity of crude extracts from cell suspension cultures of strawberry on NB and on adult colon and cervix carcinoma cell lines.

The best medium composition for cell suspension growth was detected, and the anti-proliferative activity of cell suspension extracts was determined. In order to investigate the chemical composition of suspension culture extracts, spectrophotometric, HPLC–DAD and UPLC coupled with quadrupole time-of-flight mass spectrometry (UPLC–ESI–QTOF–MS/MS) analyses were performed. The compounds were identified by the comparison of the retention time (rt) and the characteristic molecular ions and the fragment ions with those of authentic standards. When standards were not commercially available, the compounds were tentatively identified. If confirmed, the presence of certain molecules in strawberry suspension cultures, reported for the first time in this work, highlights once more the importance of plant cell culture as a source of new natural bioactive compounds.

## 2. Results and Discussion

### 2.1. Callus and Cell Suspension Culture Induction

**Figure 1:**
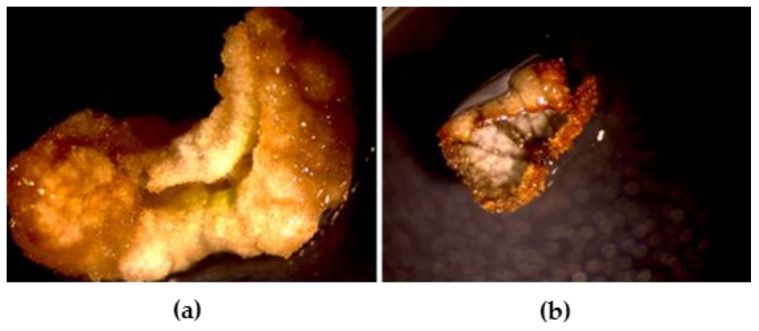
(**a**) Callus produced in GD medium; (**b**) callus induced in G medium.

For the optimization of callus induction, leaf fragments from in vitro growing shoots of strawberry were cultured on a GD medium, consisting of Gresshof & Doy basal medium [15] supplemented with 1 mg/L kinetin and 2 mg/L α-naphtalen acetic acid (α-NAA), or in G medium, consisting in Gamborg basal medium [16] supplemented with 0.1 mg/L kinetin, 2 mg/L α-NAA, 2 mg/L benzoic acid (BA), and 0.1 mg/L indole-3-acetic acid (IAA). Callogenesis was observed after 40 days (d). Calli were produced from 85% of explants. The most effective medium for callus induction, suitable for further suspension culture establishment, turned out to be GD. The callus produced in this medium was non-morphogenic, friable in appearance, and beige in color; calli induced in G medium were very compact and unsuitable for further initiation of cell suspension, thus they were not selected (Figure 1).

Light yellow and friable parts of calli from GD medium were sub-cultured every three weeks in a fresh medium, until obtaining a homogenous callus line, which was used for the establishment of cell suspension cultures in the presence of 0.2 mg/L BA and 0.5 mg/L 2,4-dichlorophenoxyacetic acid (2,4-D).

**Figure 2:**
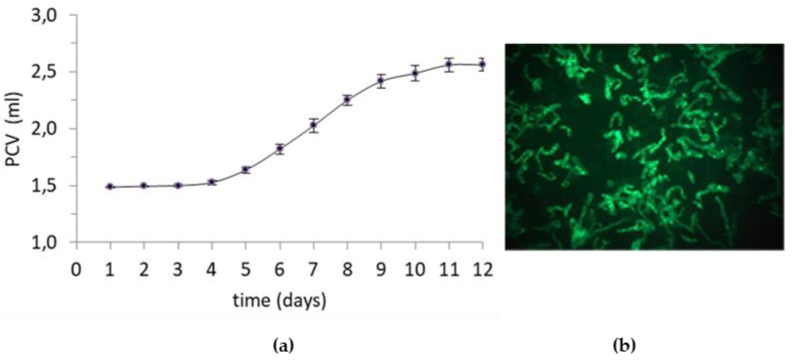
(**a**) Growth curve of cell suspension cultures of *Fragaria x ananassa* in liquid G medium, supplemented with 0.2 mg/L benzoic acid and 0.5 mg/L 2,4-dichlorophenoxyacetic acid during 12 days of culture. The bars indicate the standard error from at least three independent measurements; PCV: Packed Cell Volume; (**b**) viability of the cells stained with fluorescein diacetate examined by an epifluorescent microscope.

The cell suspension growth curve, based on Packed Cell Volume (PVC) quantification, is reported in Figure 2, panel a. After three days, the cell suspensions entered the exponential phase of culture, which was maintained for nine days. The cell growth curve and viability degree demonstrated that the cell suspensions were healthy and suitable for further investigations [17]. Suspension cultures were composed of big and small aggregates, clusters, and single cells (Figure 2b).

### 2.2. Preliminary Investigations

Extracts from cell suspension cultures were preliminary subjected to colorimetric analysis to detect the presence of polyphenols, proteins, and saponins. The yield of the extracts was around 7.8%. The mean value of total phenols content was 2.12 ± 0.13 mg gallic acid (GA) eq./100 mg dry weight (d.w.); proteins were present only in very small amounts (mean value 0.23 ± 0.04 mg bovine serum albumin eq./100 mg d.w.), and saponins were not found.

### 2.3. Chromatographic Analysis

Experiments to optimize the extraction protocol were performed. A shorter extraction time lowered the peaks area, with the maximum reached within a 24 h incubation; no signals were detected when the extractions were carried out with no acidified solvent.

**Figure 3:**
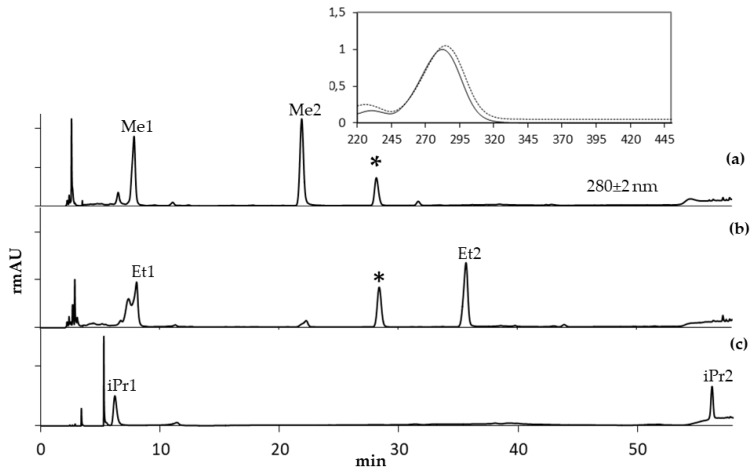
HPLC–UV chromatogram (280 nm) of lyophilized extracts dissolved in (**a**) methanol/water (70:30), (**b**) ethanol/water (70:30), (**c**) isopropanol/water (70:30). *Retention time reference (phenol). Inset shows UV–vis spectra of compounds Me1, Et1, iPr1, and 5-hydroxymethyl-2-furaldehyde (dotted line) and of compounds Me2, Et2, iPr2 (continuous line).

The chromatogram profiles of the lyophilized extracts, dissolved in three different alcohols/water (70:30) solutions, showed two main peaks at 280 nm and none relevant peaks at the other acquisition λ (not shown), indicating that phenolic acids, flavonons, and anthocyanins were poorly represented in the extracts. Electron Spray Ionization (ESI) data in negative mode further confirmed the absence of phenols. The peaks shapes and their retention times (rt_s_) did not depend on the acidification of the chromatographic eluents. The UV absorption spectra parameters of the peaks indicated their belonging to the same molecular class. Coherently with the spectrometric findings described below, Me1, Et1, iPr1, and 5-Hydroxymethyl-2-furaldehyde (2,5-HMF) coeluted, with overlapping UV absorption spectra (Table 1, Figure 3); 2,5-HMF rt did not show any alcohol-dependent significant shift.

The analysis of hydrolyzed extracts did not show any quantitative reduction of the peaks. The hydrolysis reaction led to complete sample degradation after 24 h of incubation. The HPLC profile was determined for both suspension cultures and calli and for their nutrient media. No compound was detected into the nutrient media; the callus chromatographic profile matched that of the cell culture, although a strong decrease of signal intensity was detected (not shown).

### 2.4. Spectrometric Investigation

To determine the molecular weight (MW) and the elemental composition of the compounds represented by the peaks detected by HPLC–DAD, UPLC–DAD–ESI–QTOF–MS/MS was performed. Compounds were detectable only in positive ion modes, while no signal was detected in negative ion mode. In Table 2, the accurate mass/charge ratios (*m*/*z)* of Me1, Et1, iPr1, Me2, Et2, iPr2 are reported; the molecular formulae with the corresponding MW are also reported, identified by Molecular Feature Extractor (MFE) and Molecular Formula Generator (MFG) software tools.

**Figure 4:**
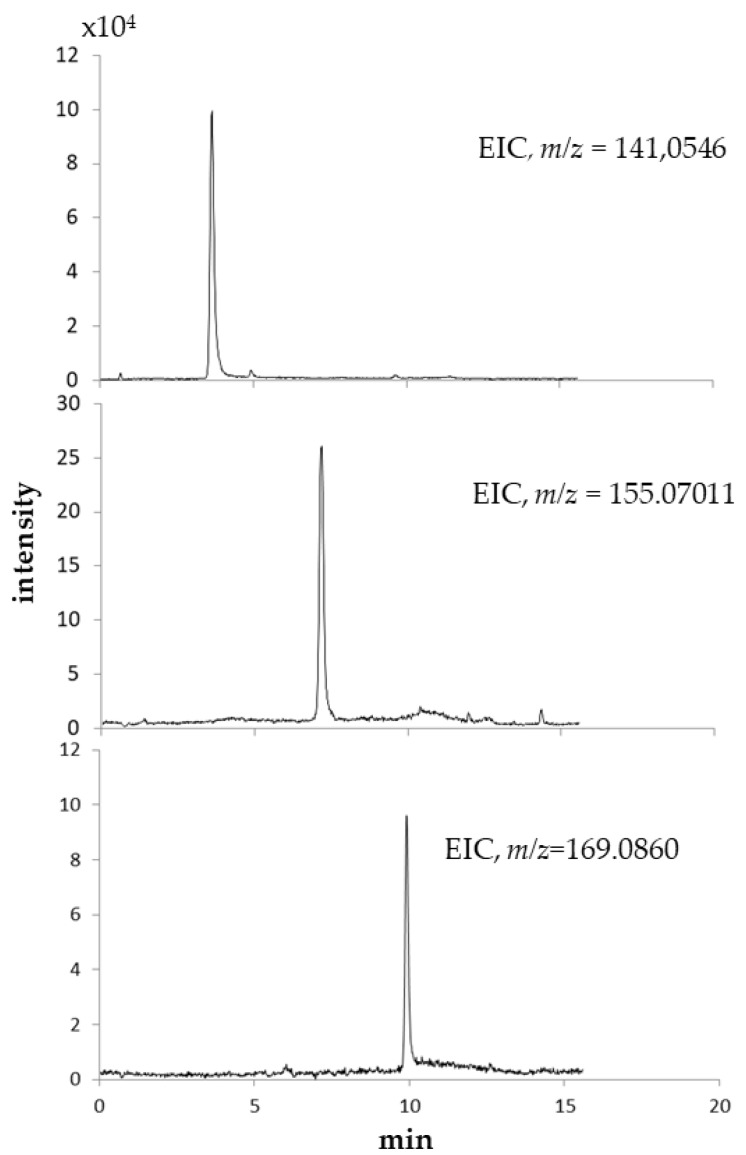
Extracted ion chromatograms (EIC) of the compounds.

For MS data, elemental composition is confirmed when mass accuracy is 5 ppm [18]; in our analyses, the error between the observed and the predicted *m*/*z* was consistently less than 1.0 ppm. In Figure 4, Extracted Ion Chromatograms (EIC) of *m*/*z* 141.0546, 155.07011, and 169.0860 are shown.

Provisional compound identification was performed by matching molecular formulae results to entries in the Chemspider database. This step resulted in a list of possible compounds; taking into account also the chromatographic data, Me1, Et1, and iPr1 were putatively identified as HMF (Table 2).

**Figure 5:**
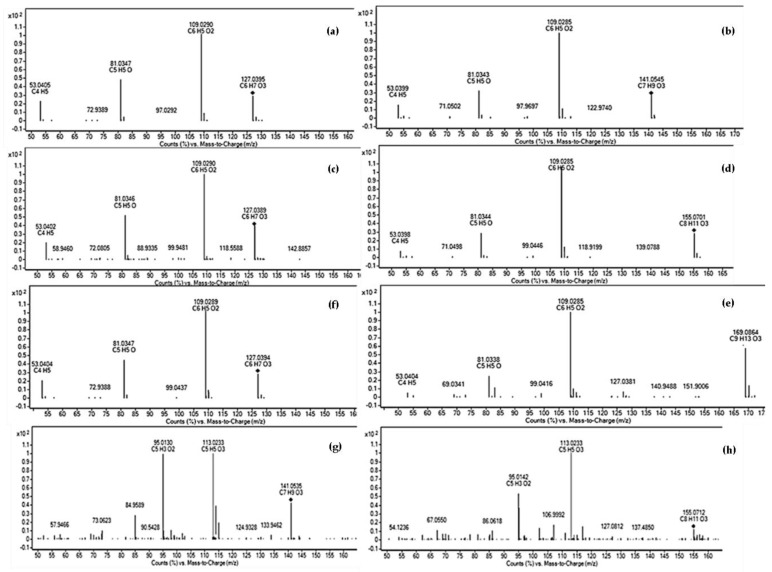
QTOF–MS/MS spectra with characteristic fragment ions of compounds (**a**) Me1; (**b**) Me2; (**c**) Et1; (**d**) Et2; (**e**) iPr2; and of standards (**f**) 5-hydroxymethyl-2-furaldehyde; (**g**) ethyl-2-furoate; (**h**) propyl-2-furoate.

QTOF–MS/MS experiments in positive mode with accurate mass measurement provided crucial structural information to suggest compound identities, allowing to assign a molecular composition to each fragment and to identify fragment losses. A common fragmentation pattern (*m*/*z* 109, 81, and 53) and fragments relative abundancies for the compounds and 2,5-HMF were found; thus, Me1, Et1, and iPr1 were definitively identified as HMF; an unstable ion at 109 *m*/*z* was present in HMF peaks, presumably due to water loss from the molecular ion. Me2, Et2, and iPr2 were tentatively deduced according to their MS/MS data. The search in a data bank for their molecular formulae reported several furanic derivates. The exclusion of furfuryl or furanoate derivates was based on the observed chemical, chromatographic, and spectrometric behaviors (sensitivity to acid hydrolysis, UV spectra and rt_s_, MS/MS fragmentation patterns) (Figure 5, Table 3).

**Figure 6:**
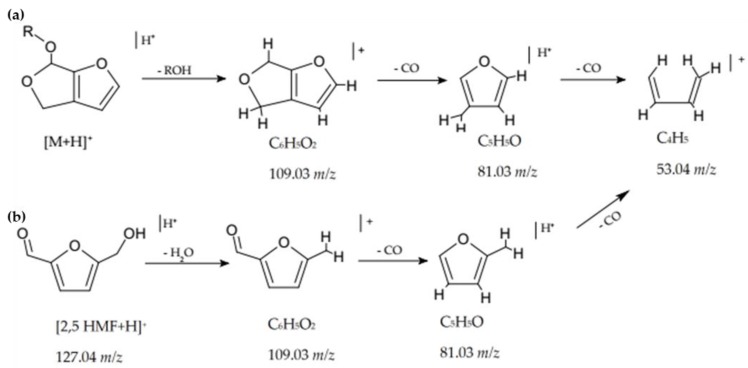
Proposed chemical structures, fragmentation pathways, and characteristic fragmentation ions of: (**a**) compounds in the extracts (R = Me, MW = 140; R = Et, MW = 154; R = iPr, MW = 168); (**b**) hydroxymethyl-furaldehyde (HMF).

The MW of Me2, Et2, and iPr2 compounds and the calculated molecular formulae showed a progressive increase of –CH2 units; similarly, the rt_s_ of Me2, Et2, and iPr2 shifted to higher values when the extracts were dissolved in alcohol solutions, according with the homologous series (Table 1, Figure 3). On the basis of the results coming from the investigations above reported [19], we then could propose a dihydrofurofuran (DHFF) acetal class compound and its fragmentation pathway, where *m*/*z* 109 corresponds to the neutral loss of the alcoholic chain (Figure 6a) or to the loss of a molecule of water in the case of HMF (Figure 6b), while *m*/*z* 81 and 53 correspond to the neutral loss of CO molecules. Unfortunately, the complete identification of Me2, Et2, and iPr2 was not possible because of the lack of reference compounds.

**Figure 7:**
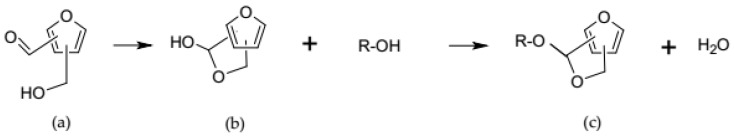
Cyclization of the hydroxyl group with the vicinal aldehyde group and acetylation with alcohol. (R = Me, MW = 140; R = Et, MW = 154; R = iPr, MW = 168). (**a**) vic HMF; (**b**) vic HMF emiacetal; (**c**) vic HMF acetal.

We speculated about the presence, in the lyophilised extracts, of a highly reactive furanic compound that, once dissolved, binds alcohol through an addiction reaction. The reaction occurred rapidly and quantitatively, at room temperature (area %, Table 1). Among the organic molecules with this reactivity, both aldehydes and acids (producing acetals and esters, respectively) were considered. The furanic acids class was excluded, since no furoate fragmentation patterns occurred. The reactivity of 2,5-HMF with alcohols was also excluded, while an HMF molecule having the hydroxyl close to the aldehyde group could undergo a cyclization reaction, leading to hemiacetal formation and subsequent acetylation with alcoholic solvent (Figure 7).

We argued that HMF detected in the acid alcoholic extract could be the product of the degradation of cell wall constituents, as it is known that the sugar components present within cellulose and hemicellulose can be degraded to hydroxy-methyl-furfural [20,21]. Furofurans (FF) are present in plants as precursor of lignans, and some lignans have been previously found in strawberry [22,23,24,25]. The DHFF acetals observed in this work could probably come from lignan pathway perturbations occurring in in vitro conditions, from the extraction procedures, or both.

### 2.5. Cytotoxic Effects of Extracts on Cancer Cell Lines

**Figure 8:**
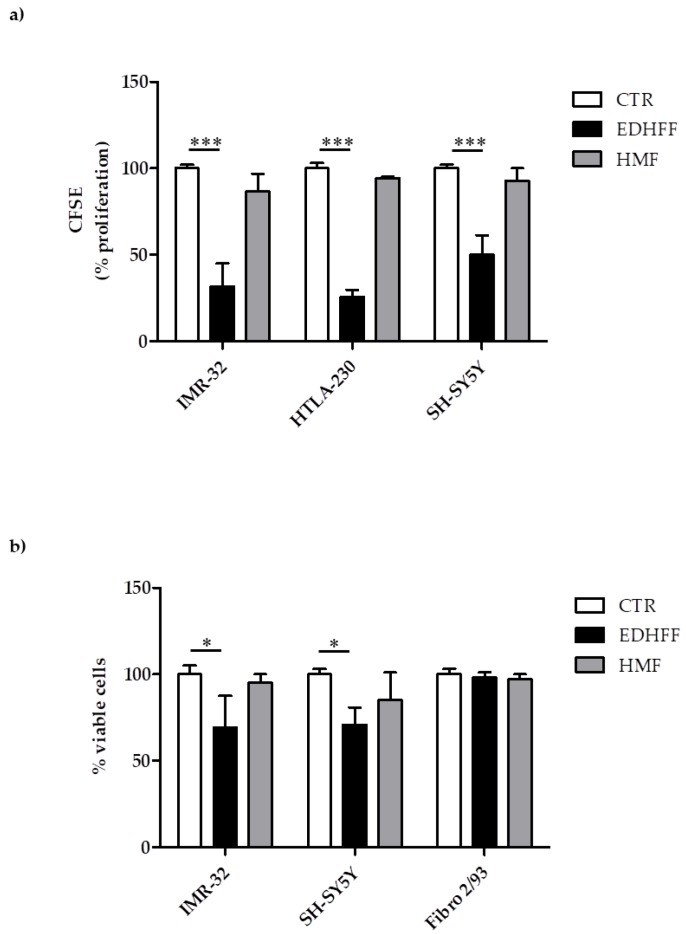
Effect of ethoxy-dihydrofuro-furan (EDHFF, 0.075 mM) from strawberry cell suspensions extracts (**a**). Human neuroblastoma (NB) cell lines proliferation; (**b**) viability of NB and human fibroblast (from healthy donors, Fibro 2/93) cell lines. CTR: solvent control cells incubated with the solving solution (ethanol/water, 70:30) Data expressed as means ± SE, * *p* < 0.05; *** *p* < 0.001: EDHFF vs. CTR.

The cell lines were treated with extracts dissolved in a solution of ethanol/water, 70:30 (*v/v*), (EtOH 70%), where the presence of the ethoxy-DHFF acetal (EDHFF) and of HMF (compound Et2 and Et1, respectively) was previously shown (Table 3). The cytotoxic potential of EDHFF was firstly evaluated in human NB cell lines in terms of inhibition of cell proliferation and decrease of cell viability. Both MYCN-amplified (IMR-32 and HTLA-230) and MYCN-single-copy (SH-SY5Y) NB cells treated with sample extracts containing 0.075 mM EDHFF showed a significant inhibition of proliferation, when compared to control (CTR) cells. Interestingly, pure HMF (15 µg/mL) used at the highest concentration detected in the sample extracts did not shown any effects on NB cells. Since no other molecular class could be quantitatively detected in the extracts, at least under our experimental conditions, these findings indicated that the inhibition of cell proliferation was due solely to EDHFF. Moreover, the EDHFF-driven anti-proliferative effect translated into a significant decrease of NB cells viability. Of note, human fibroblasts from healthy donors (Fibro 2/93) were not affected (Figure 8).

**Figure 9:**
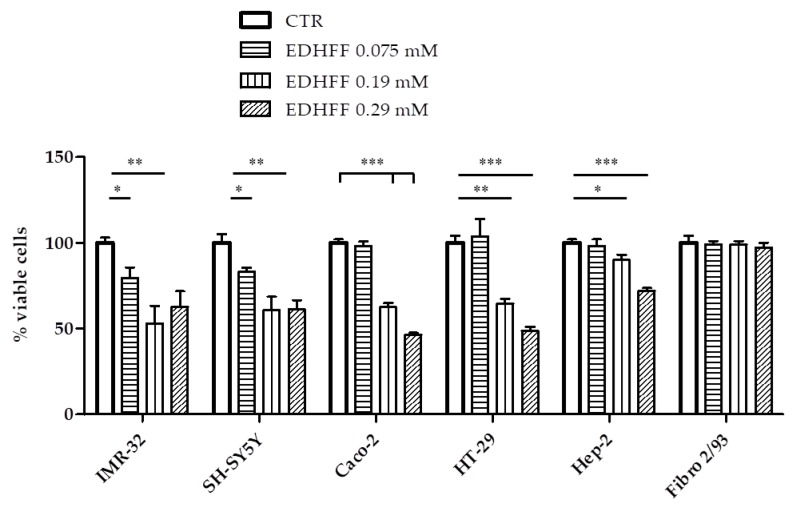
Effect of EDHFF (0.075 mM, 0.19 mM, and mM 0.29) from strawberry cell suspensions extracts on the viability of human neuroblastoma (IMR-32, SH-SY5Y), colorectal adenocarcinoma (Caco-2), colon adenocarcinoma (HT-29), and cervix carcinoma (Hep-2) cell lines. CTR: solvent control cells incubated with the solving solution (ethanol/water, 70:30). Data expressed as means ± SE, * *p* < 0.05; ** *p* < 0.01; *** *p* < 0.001: EDHFF vs. CTR.

In a second set of experiments, the cytotoxic effects of extracts containing different amounts of EDHFF were tested on paediatric and adult human tumor cell lines. Figure 9 clearly shows that all cells were sensitive to EDHFF. A concentration-dependent cytotoxic effect of EDHFF was exerted on NB and adult tumors at concentrations from 0.075 to 0.019 mM (followed by a response plateau) and from 0.19 to 0.29 mM, respectively. Again, Fibro 2/93 were not affected by EDHFF at any concentration used, indicating the importance of further preclinical investigations.

Fused polycyclic acetals are contained in a broad range of natural products. Among bicyclic acetals, FF derivatives are of special interest, since their biological and pharmaceutical activities are known [26]; however, their role as antitumor compounds is still controversial and needs to be elucidated [27]. The DHFF unit is an important skeletal structure in various biologically and pharmaceutically active organic molecules and it is very useful for organic transformations to potential bioactive molecules [28]. As the isolation or the preparation of different types of FF lignans is difficult, the analysis of the relationship between the FF ring, its conjugates, and the established clinical activities has not been done yet [20,29,30,31].

To our knowledge, this is the first manuscript that reports the direct effect of an aglycone form of the FF skeleton on its anti-proliferative activity.

## 3. Materials and Methods

### 3.1. Chemicals and Reagents

All reagents and solvents were purchased by Sigma-Aldrich, Italy, except when differently specified. Chromatography chemicals were of HPLC or LC/MS grade. Ultrapure water (18.2 MΩ) was daily prepared with a Milli-Q water purification system (Millipore, Bedford, MA, USA).

### 3.2. In Vitro Shoot Cultures 

Axillary buds of in vivo growing strawberry (*Fragaria x ananassa* Duch.), cv. Don, from the germplasm collection of the CREA-OFA (Rome), were sterilized by immersion in EtOH 70% for 1 min, in commercial bleach solution (1% *w/v* sodium hypochlorite) for 20 min, rinsed with sterile deionized water, and cultured on Quoirin & Lepoivre (QL) macrosalts [32], Murashige & Skoog (MS) microsalts and organics [33], 30 g/L sucrose (Eridania, Parma, Italy), 5.5 g/L agar (B & V– Reggio Emilia, Italy), 0.3 mg/L of BA, 0.1 mg/L indole-3-butyric acid, and 0.1 mg/L of gibberellic acid. Shoots were sub-cultured every 21 d. Growth chamber standard conditions were 24 ± 1 °C, 16 h photoperiod with a light intensity (fluorescent tubes Philips TLD 58W/33) of 25 µmol m^2^ s^−1^ photosynthetic photon flux (PPF).

### 3.3. Establishment of Calli and Cell Suspension Cultures

To induce callus formation, leaf explants of the in vitro strawberry growing shoots were cut into small pieces and aseptically transferred, adaxial side down, into GD medium consisting of Gresshof & Doy salts and organics [15], supplemented with 1 mg/L kinetin and 2 mg/L α-NAA, or in G medium consisting of Gamborg salts and organics [16] supplemented with 0.2 mg/L kinetin, 2 mg/L α-NAA, 2 mg/L BA, 0.1 mg/L IAA. Sucrose (20 g/L) and 5.5 g/L agar were added to both media. Cultures were maintained in a growth chamber (24 ± 1 °C), in darkness, for the first two sub-cultures and then transferred to the light. Callus sub-cultures were carried out every 21 d. Three Petri dishes containing 20 leaf explants for each treatment were used to induce callus formation. After one sub-culture in the light, calli produced from leaf explants grown in GD medium were used for establishing the cell suspension cultures.

To this aim, calli (approximately 1 g) were transferred to 10 mL liquid medium consisting of [16] salts and organics and sucrose (20 g/L), supplied with 0.2 mg/L BA and 0.5 mg/L 2,4-D, and incubated on an orbital shaker (90 rpm). cultures were maintained on an orbital shaker (90 rpm) (Cavallo, Milan, Italy) in growth chamber standard conditions and sub-cultured every 10 d during the exponential (log) phase. Cell growth was determined every day during a 14-day period, by measuring the PCV, i.e., the volume of cells pellet after 10 min centrifugation (1500× *g*); the initial concentration of the cell cultures was 1 mL PCV in 7 mL of liquid medium [34]. All measures were performed at least in triplicate. The determination of cell viability was performed by observation of the cells stained with FDA under an epifluorescent microscope (Leitz-Fluovert, Wetzlar, Germany) [35].

### 3.4. Suspension Cultures Extraction

The cells were collected during the exponential (log) phase of the third sub-culture (10th day), rapidly frozen in liquid nitrogen (LN), and stored at −80 °C until extraction. The extraction was optimized from pilot experiments. The concentration of hydrochloric acid and the extraction time (from a few hours to several days) were determined by measuring the maximum chromatographic total peak area (%). These optimal conditions were then applied as follows: each sample (50 mg) was homogenized in LN and extracted in methanol/water 70:30 (*v/v*), solvent solution (MeOH 70%), 1% HCl. The samples were sonicated in an ultrasonic water bath (frequency 28.5/31 Khz, power 400 W) for 10 min. After incubation at 4 °C for 24 h in darkness, the mixtures were centrifuged at 3000× *g* for 10 min. The pellet was extracted again, and the two supernatants were combined. The combined supernatants were placed in a lyophilizer for 24 h at −40 °C with a pressure of 6 × 10^−2^ mbar (Edwards Freeze Dryer Modulyo, Crawley, England) and stored at room temperature until analysis.

The extraction yield was expressed as:

Extraction yield (%) = Weight of the dry extracts (g)/Weight of the dry cells (g) × 100(1)

The lyophilized extracts were accurately weighed and dissolved in MeOH 70%, EtOH 70%, and isopropanol/water 70:30 (*v/v*) (*i*PrOH 70%), then centrifuged for 15 min at 10,000× *g*. After centrifugation, the supernatant was collected, filtered through a 0.22 μm filter (PTFE syringe filter), and immediately analyzed or stored at −20 °C until further analysis. All the procedures were carried out under dim light, and glassware containing the samples were covered with aluminium foil to minimize photooxidation. 

### 3.5. Preliminary Spectrophotometric Assays

The spectrophotometric analyses reported below were performed on a Hitachi U-2000UV–visible spectrophotometer. TP content was spectrophotometrically determined according to [36], using the Folin–Ciocalteu method with slight modifications. Briefly, 10 µL of the extract was transferred into a test tube, and 50 µL of Folin–Ciocalteau reagent was added. Then, 100 µL of 20% (w/v) sodium carbonate was added, and the sample was mixed thoroughly. The final reaction volume was 1 mL. After 20 min in darkness, the absorbance was measured at 700 nm. For the standard calibration curve (50 to 300 mg/L), GA was used with a seven-point calibration curve (R^2^ > 0.99). The results were expressed as mg GA equivalent per 100 mg of d.w. (mg CAeq/100 mg d.w.). Standard Bradford method was used to detect the presence of proteins [37] expressed as mg of bovine serum albumin (BSA) equivalent per 100 mg of d.w. (mg BSA eq/100 mg d.w.). Absorbance was measured at 595 nm. For the standard calibration curve (0.05 to 0.283 mg/L), BSA was used, with a four-point calibration curve (R^2^ > 0.99). The Zlatkis method, with slight modifications, was used to detect the presence of saponins [38]. Absorbance was measured at 545 nm, and cholesterol was used as a standard (1.0 and 0.5 mg/mL) [39].

Each analysis was performed on three samples and repeated twice.

### 3.6. Acid Hydrolysis 

An aliquot (1 mL) of sample was added to 200 μL of 2N HCl (final concentration), and the solution was thoroughly mixed. Part of the solution (500 µL) was maintained at 85 °C for 2 h and was then immediately cooled in ice. The remaining solution was maintained at 85 °C overnight.

### 3.7. Instrumentation and Chromatographic Conditions

The samples were analyzed in reverse phase by HPLC–DAD (Agilent 1100 UV–Visible, Milan, Italy) with a ZORBAX SB-C18, 5 μm column, id 4.6 mm, and length 250 mm (Agilent), without consideration of any specific group of compounds. A gradient solvent elution system was used over 75 min with the following eluents: (A) H_2_O + 0.5% (*v/v*) acetic acid, 1% (*v/v*) H_3_PO_4_, (B) acetonitrile + 0.5% (*v/v*) acetic acid, 1% (*v/v*) H_3_PO_4._ The same solvent elution system, except for eluents acidification, was also applied. The solvent gradient was as follows: 5% B at 0–5 min, 5–15% B at 5–30 min, 15–20% B at 30–35 min, 20% B at 35–40 min, 20–25% B at 40–50 min, 25–90% B at 50–60 min, 90% B at 60–75 min. The flow rate was 1 mL/min. The sample injection volume was 20 μL. The runs were monitored at 280, 320, and 510 nm. Phenol was used in order to have a retention time reference. The retention times and UV spectra of the peaks in the samples were compared with those of authentic standards or to data reported in the literature.

For quantitative analysis, we used the external standard method. We assumed the same correction factor for 2,5-HMF (ChemSpider ID: 207215) and all identified compounds. A three-level calibration curve was obtained by the injection of known concentrations (61–32 µg/mL) of 2,5-HMF. The results were expressed as mg HMF/mL.

UPLC analysis was performed by using an Agilent 1290 Infinity Binary LC with UV–VIS photodiode array detector (DAD G4212A) coupled with an Agilent 6530 Accurate Mass Quadrupole Time-of-Flight, equipped with a dual spray ESI source, which provides a resolution of 5000 to 10,000 (100 to 922 *m*/*z*), a mass accuracy error less than 5 ppm, and a detection frequency of 2 GHz in Extended Dynamic Range state. The system was controlled by an Agilent MassHunter workstation software (version number B.06.00, Agilent 182 Technologies, Inc. 2011). Separation was performed on a Zorbax Eclipse Plus C18 RRHD column (2.1 × 50 mm, 1.8 μm, Agilent) at 25 °C. The eluents consisted of water (A) and acetonitrile (B), both acidified with formic acid 0.1% (*v/v*). All samples were eluted under the following conditions: isocratic conditions with 5% B from 0 to 1 min; linear gradient conditions from 5% to 15% B for 5 min; from 15% to 20% B for 1 min; isocratic with 20% B for 1 min; linear from 20% to 90% B for 4 min; isocratic with 90% B for 3 min; post-time 1 min). The flow rate was 0.21 mL/min, and the injection volume was 0.80 μL.

Total ion spectra were collected over a mass range of *m*/*z* 80−1,100 (scan rate 1.00 spectra/sec), in both negative and positive modes. The values of the ESI–MS parameters in ESI source positive and negative ionization mode were as follows: drying gas (N_2_) flow rate, 8.0 L/min; drying gas temperature, 300 °C; Nebulizer, 20 psig; capillary voltage, 3000 V; fragmentor 175 V; skimmer voltage, 60V; octupole RF peak 750 V. The quadrupole temperature was set at 100 °C. The mass axis was calibrated using an external reference mass solution (121.0509 *m*/*z* and 922.0098 *m*/*z*, positive mode; 68.9958 *m*/*z*, 112.9856 *m*/*z*, and 1033.9881 *m*/*z*, negative mode), which were provided by the manufacturer (Agilent), prepared in acetonitrile. All data were collected in centroid mode. 

Precursor ions of interest ([M + H]^+^) were selected and then subjected to collision at multiple fragmentation energies (5 eV, 10 eV, 20 eV). The masses selected for MS/MS analysis are reported in Table 4.

The collision gas was nitrogen (purity 99.999%). The data were collected by targeted MS/MS acquisition with an MS/MS scan rate of 4.00 spectra/sec, mass range 50 *m*/*z*–1000 *m*/*z*. Daughter ion spectra were compared with data produced for the standards under the same instrumental conditions. For qualitative analysis, 32.0 μg/mL of 2,5-HMF, 5.64 mg/mL of 5-ethyl-2-furoate (MW 140.047348, Chemspider ID:11485), and 4.58 mg/mL propyl-2-furoate (MW 154.062988, Chemspider ID: 11487) were accurately weighed and dissolved in MeOH 70% and EtOH 70% to give individual standard stock solutions.

### 3.8. Data Analysis

The Agilent Mass Hunter Workstation-Qualitative Analysis software (ver. B.06.00) was used for the initial processing of the UPLC–ESI–QTOF–MS data. Software tools were used to aid in the identification of the compounds, including the MFE and MFG algorithms. Compounds were identified from the raw data, in a fully automated mode, assuming C, H, N, O, S and [M + H]^+^ as pseudomolecular ions to generate the elemental formulae. Provisional compound identification was performed by matching molecular formulae results to entries in the Chemspider database.

The comparison of orthogonal information from samples and standards was used for putative metabolite identification and molecular structure elucidation [19].

Chemical nomenclature was generated by ACD/ChemSketch^®^. 

### 3.9. Cell Lines and Strawberry Extract Treatments

MYCN-amplified (HTLA-230 and IMR-32) and MYCN-single-copy (SH-SY5Y) human NB cell lines, representing the genomic and phenotypic variability of NB, and human fibroblasts from healthy donors (Fibro 2/93) were grown in complete Dulbecco’s Modified Eagle Medium (DMEM) or RPMI-1640 medium, as previously described [40]. For adult human tumor cell lines, epithelial HEp-2 cell line (ATCC^®^ CCL23™), colon adenocarcinoma HT-29 cell line (ATCC^®^ HTB-38^™^), and colorectal adenocarcinoma Caco-2 (ATCC^®^ HTB-37^™^) cell line were grown in DMEM supplemented with 10% fetal bovine serum (Flow Laboratories, Rockville, MD, USA), 5 mM lL-glutamine, 100 U/mL penicillin, and 100 μg/mL streptomycin. The cells were tested for mycoplasma contamination and characterized in terms of cell proliferation and morphology.

In the cytotoxic experiments, the cells were seeded at a density of 2.5–3 × 10^5^, 1.5 × 10^5^, and 2 × 10^5^ cells/cm^2^ for NB, Fibro 2/93, and adult tumor cell lines, respectively, and, 24 h after, exposed for 48 h to the strawberry extracts, dissolved in EtOH 70%. EDHFF final concentration corresponded to 0.075 mM, 0.19 mM, and 0.29 mM (± 10% for each value). Viable cells were then counted on the basis of Trypan blue exclusion.

In the carboxyfluorescein succinimidyl ester (CFSE) proliferation assay, NB cells were incubated the day before with CellTrace™ CFSE staining solution (2 µM) for 15 min at 37 °C degrees, following the manufacturer’s instruction (Thermo Fisher Scientific). After 24 h, the labeled cells were treated with 0.075 mM (± 10% for each value) EDHFF (final concentration). CFSE dilution was evaluated by FACS analysis. 

Cancer cell lines treated with pure HMF were included as a control, at HMF concentration of 15 µg/mL, which represents the highest HMF concentration detected in the sample extracts.

Each treatment was performed on three samples and repeated at least three times.

### 3.10. Statistical Analysis

The standard error (SE) was calculated on the data collected, and significant differences among mean values were assessed with Prism 5 software (GraphPad, La Jolla, CA, USA). One-way analysis of variance (ANOVA) with Tukey’s Multiple Comparison Test was used to evaluate differences within treatments. Asterisks indicate the following *p*-value ranges: * = *p* < 0.05, ** = *p* < 0.01, *** = *p* < 0.001.

Each analysis was performed on three samples per extract and repeated at least three times. 

## 4. Conclusions

An efficient cell suspension culture was established with leaves-derived friable calli of micropropagated strawberry (*Fragaria x ananassa* Duch), cv. Don, as a prerequisite for in vitro studies of the production of bioactive metabolites. Extracts from the in vitro cell suspension cultures induced a statistically significant reduction of proliferation in all cancer cell lines tested. Strawberry healthy effects have been mainly attributed to polyphenols, though this conclusion is still controversial. In fact, the evidence supporting the potential anti-proliferative effects of strawberry polyphenols in several types of cancer in humans is still very limited when compared with that obtained in preclinical studies.

Here, we propose the novel molecule EDHFF from extracts of in vitro cell suspension culture of strawberry as an anti-proliferative agent. More research is needed to evaluate, in molecules of the DHFF class, the effects on the cytotoxic activity of the alkoxy chain in relation to its length (i.e., methoxy-DHFF and isopropoxy-DHFF). The data provided by the current study warrant NMR approaches to confirm the identity of the proposed molecule, and further studies are needed to investigate the mechanisms of the anti-proliferative effects of the putative compound and to optimize its productive process and purification. Our results confirm the potential of in vitro cultures as drug factories for the production of novel compounds for pharmaceutical studies. The discovery of compounds that are, at present, unidentified suggests that bioactive chemicals derived from plants can be detected via the proposed in vitro culture strategy, which represents a simple approach to finding novel useful metabolites.

## Figures and Tables

**Table 1:** Peaks retention times (rt, min), areas (%), and absorbance spectra parameters (λ_max_ and match value).

Solvent (70:30)	Peak Name	rt (min)	Area (%)	λ_max_ (nm)	Match
MeOH/H_2_O	Me1	8.47	51.23	284	999.618
	Me2	21.85	48.77	282	984.860
EtOH/H_2_O	Et1	8.05	51.41	284	999.970
	Et2	35.70	48.59	282	989.726
*i*PrOH/H_2_O	iPr1	6.20	57.15	284	999.918
	iPr2	56.26	42.85	282	983.303
MeOH/H_2_O	HMF	8.63		284	

MeOH: methanol; EtOH: ethanol; *i*PrOH: isopropanol; HMF: 5-hydroxymethyl-2-furaldehyde.

**Table 2:** Compounds determined by UPLC–Q–TOF/MS; amu: atomic mass unit.

Solvent (70:30)	Peak	Detected Mass [M + H]^+^ (amu)	Calculated Mass (amu)	Molecular Formula	Molecular Mass (amu)	Error (ppm)
MeOH/H_2_O	Me1	127.0389	126.0317	C_6_H_6_O_3_	126.0317	−0.16
	Me2	141.0546	140.0473	C_7_H_8_O_3_	140.0474	−0.11
EtOH/H_2_O	Et1	127.0391	126.0317	C_6_H_6_O_3_	126.0318	−0.85
	Et2	155.07011	154.0630	C_8_H_10_O_3_	154.0629	0.91
*i*PrOH/H_2_O	iPr1	Not detectable				
	iPr2	169.0860	168.0786	C_9_H_12_O_3_	168.0786	−0.7
	HMF	127.0389	126.0317	C_6_H_6_O_3_	126.0316	0.74

**Table 3:** MS/MS fragments (collision energy 5 ev) and proposed assignment.

Compound	Rt (min)	Characteristic Fragmentation Ions (*m*/*z*)	Molecular Formula	Error (ppm)	Assignment
		and Relative Abundances			
Me1	1.9	[127→]: 127.0395 (10.08)	C_6_H_7_O_3_	−3.97	HMF
		109.0290 (100.00)	C_6_H_5_O_2_	−5.23	
		81.0347 (47.96)	C_5_H_5_O	−14.99	
		53.0405 (22.77)	C_4_H_5_	−36.64	
		[109→]*: 109.0285 (100.00)	C_6_H_5_O_2_	−0.86	
		81.0343 (96.52)	C_5_H_5_O	−9.98	
		53.0398 (80.58)	C_4_H_5_	−23.07	
Me2		[141→]: 141.0545 (26.14)	C_7_H_9_O_3_	0.66	Methoxy-
		109.0285 (100.00)	C_6_H_5_O_2_	−0.96	dihydrofuro-furan
		81.0343 (32.01)	C_5_H_5_O	−9.72	
		53.0399 (15.55)	C_4_H_5_	−24.68	
Et1	1.66	[127→]: 127.0389 (38.43)	C_6_H_7_O_3_	0.49	HMF
		109.0290 (100.00)	C_6_H_5_O_2_	−5.75	
		81.0346 (51.82)	C_5_H_5_O	−13.75	
		53.0402 (19.61)	C_4_H_5_	−30.57	
		[109→]*: 109.0299 (82.29)	C_6_H_5_O_2_	−13.38	
		81.0346 (100.00)	C_5_H_5_O	−13.77	
		53.0410 (50.08)	C_4_H_5_	−46.38	
Et2	7.18	[155→]: 155.0701 (28.25)	C_8_H_11_O_3_	0.92	Ethoxy-
		109.0285 (100.00)	C_6_H_5_O_2_	−1.28	dihydrofuro-furan
		81.0339 (28.73)	C_5_H_5_O	−10.62	
		53.0398 (7.65)	C_4_H_5_	−22.33	
iPr2	9.9	[169→]: 169.0864 (57.32)	C_9_H_13_O_3_	−2.97	Isopropoxy-
		109.0285 (100.00)	C_6_H_5_O_2_	−0.94	dihydrofuro-furan
		81.0338 (24.72)	C_5_H_5_O	−4.25	
		53.0404 (5.19)	C_4_H_5_	−34.02	
Standard Compounds					
2,5-HMF	1.91	[127→]: 127.0394 (27.64)	C_6_H_7_O_3_	−3.63	
		109.0289 (100.00)	C_6_H_5_O_2_	−4.74	
		81.0347 (44.59)	C_5_H_5_O	−14.63	
		53.0404 (20.76)	C_4_H_5_	−34.85	
		[109→]*: 109.0292 (100.00)	C_6_H_5_O_2_	−7.02	
		81.0348 (87.99)	C_5_H_5_O	−16.19	
		53.0403 (83.09)	C_4_H_5_	−32.78	
Ethyl-2-furoate	10.39	[141→]: 141.0535 (42.94)	C_7_H_9_O_3_	7.67	
		113.0233 (100.00)	C_5_H_5_O_3_	0.42	
		95.0130 (99.29)	C_5_H_5_O_2_	−2.63	
Propyl-2-furoate	11.38	[155→]: 155.0712 (12.54)	C_8_H_11_O_3_	−6.13	
		113.0233 (100.00)	C_5_H_5_O_3_	0.25	
		95.0142 (53.88)	C_5_H_5_O_2_	−14.94	

* unstable ion; HMF: Hydroxymethyl-furaldehyde.

**Table 4:** Masses selected for MS/MS analysis.

Targeted Mass *	Rt (min)
109	1.50
127	1.50
141	3.63
155	7.18
169	9.92

* Isolation width: 4 amu.
